# Are Lipids Possible Markers of Suicide Behaviors?

**DOI:** 10.3390/jcm10020333

**Published:** 2021-01-18

**Authors:** Agnieszka Kułak-Bejda, Grzegorz Bejda, Magdalena Lech, Napoleon Waszkiewicz

**Affiliations:** 1 Department of Psychiatry, Medical University of Bialystok, 16-070 Choroszcz, Poland; ml.magdalenalech@gmail.com (M.L.); napoleonwas@yahoo.com (N.W.); 2 Faculty of General Medicine, School of Medical Science in Bialystok, 15-875 Bialystok, Poland; grzegorzbej@gmail.com

**Keywords:** suicide, lipids, cholesterol, markers

## Abstract

Suicides and suicidal behaviors are very important causes of mortality and morbidity and have become a serious global problem. More than 800,000 people die from suicide every year. Previous researches have established that lipids play an important role in the pathogenesis of suicide. Moreover, lipid levels might be a biological marker of suicide. A lot of researchers have tried to identify biological markers that might be related to depressive disorder, bipolar disorder or schizophrenia and suicidal behavior. It was also important to consider the usefulness of an additional tool for prevention actions. Metabolic deregulation, particularly low total cholesterol and low-density lipoproteins-cholesterol levels may cause higher suicide risk in patients with these psychiatric disorders.

## 1. Introduction

Suicides and suicidal behaviors are very important causes of mortality and morbidity and have become a serious global problem. Over 800,000 people die from suicide every year [1,2]. Suicide is one of the primary causes of death worldwide [3,4,5]. Moreover, it is one of the most devastating consequences of psychiatric disorders [6,7]. Suicide attempters are not a homogeneous group. There are many differences in their psychopathology, risk factor profiles, neurobiology, and neurochemistry; for example, oxidative stress plays an essential role in the pathophysiology of suicide, as do changes in brain function. These differences were observed via functional magnetic resonance imaging (fMRI) [8,9,10]. It is proved that depression and psychosis intensify suicidal ideation and suicide [11].

Many studies establish that lipid changes may be related to suicidality. There is a theory that serum lipid levels play an essential role in the pathogenesis of suicide [3,12,13,14]. A relationship between low serum cholesterol levels and suicide in patients with, e.g., depression, early psychosis, impulsivity, or violent behavior was established. [9,15,16,17].

One possible explanation is the association of monoamine oxidase (MAO) theory. The experimental studies demonstrated increased MAO A and MAO B activity in the hippocampus of mice [18,19]. Another theory assumes that lower cholesterol level may decrease membrane fluidity and cause an increase in the order of the membrane lipid bilayer. This might decrease serotonergic neurotransmission.

Cholesterol is a vital constituent of cellular membranes and plays an important role in membrane function. It was found dispersed in a non-random form in domains in membrane models [6,20,21,22]. These areas are assumed to be essential in the protection of the structure and function of the membrane. It has been indicated that these formations are membrane domains in which signalling from a neurotransmitter may arise like serotonin1A (5-HT1A) receptor [6,23,24].

Researchers demonstrated the major role of membrane cholesterol in the function of the 5-HT1A receptor [6,25,26,27,28,29,30]. It has been shown that the fluidity of lipids significantly controls the binding of serotonin (5-HT) in animal brain membranes. It is believed that lower levels of cholesterol increase the fluidity of cellular membrane. Alternatively, minimal exposure of the 5-HT receptors would be detected in the synaptic cleft [6,31,32].

It was shown that the percentage of suicide is three times higher in individuals with first-episode psychosis compared to the general population. Furthermore, suicide rates are higher throughout the progression of schizophrenia [9,33,34,35,36]. It has been demonstrated that 8.6% of suicidal behaviors occurred in patients with psychosis before their first visit to a hospital and 5.3% during the first year of treatment [9,13,14].

Furthermore, the severity of depression and bipolar disorder and are accompanied by a high suicidal risk [9,12,22,37].

Moreover, decreased blood levels of cholesterol have been related to violent [38,39] and aggressive behavior [40].

The aim of this study was to present the possible role of lipids as biological markers of suicide in adults.

## 2. Materials and Methods

We present an overview of the literature from the main databases (Medline, Web of Science, EMBASE, and the Cochrane Database of Systematic Reviews). We restricted our search to studies published in the last five years. Only English-language articles were included. We searched for the following terms: “cholesterol” OR “total cholesterol” OR “low-density lipoprotein” OR “LDL” OR “very low-density lipoprotein” OR “VLDL” OR “high density lipoprotein” OR “HDL” OR “triglycerides” OR “TG” and “suicide”. Review articles and case reports were excluded. We also limited our research to adult samples.

## 3. Results

### 3.1. Total Cholesterol

For adults, total cholesterol levels must be lower than 200 mg/dL. The 220−239 mg level range is considered borderline high, and a reading of 240 mg/dL and above is thought high [41].

Bartoli et al. [42] found that persons who had attempted suicide had levels of total cholesterol (TC) (174.0 ± 45.7 mg/dL) lower than those who had not attempted suicide (193.9 ± 42.6 mg/dL) (*p* = 0.004). This finding was observed in violent (174.1 ± 46.2 mg/dL) as well as non-violent (173.8 ± 46.1 mg/dL) persons who had attempted suicide (*p* = 0.035 and 0.016, respectively).

Other researchers have observed that total esterified cholesterol measured at the time of psychiatric assessment shared a significant genetic similarity with the risk for attempting suicide (ρg = −0.64, *p* = 1.24 × 10^−04^). They confirmed relationship between total cholesterol and suicidal risk was significantly mediated by ABCA-1-specific cholesterol efflux capacity [43].

Aguglia and co-workers [44] conducted a study with a sample of 632 attempts. They demonstrated that those who attempted high-lethality suicide had significantly lower total cholesterol. In another study, Aguglia et al. [45] focused on the predictors of relapse in the same group of individuals and noticed a correlation between lower TC serum levels and repeated suicide attempts [45]. 

In 2017, Messaoud and co-workers [36] performed research on Tunisian patients diagnosed with major depressive disorder (MDD). They observed a substantial decrease in plasma cholesterol levels in the group of suicidal depressive patients compared to controls. 

Similar conclusions were reached by Segoviano-Mendoza et al., who found a significant decrease in total cholesterol serum levels in patients with MDD and suicide attempts compared to those without suicidal behavior. Moreover, lower cholesterol concentrations were significantly correlated with MDD and suicide attempts [6]. Chen et al. also found that the risk of suicidal death increased with lower serum TC levels across the three years preceding death by suicide [46].

Svensson et al. presented interesting findings resulting from their prospective study based on the Japan Public Health Center (JPHC) study from 1990 to 2012 [18]. This study demonstrated that high TC levels are related with an almost twofold increased risk of suicide in women in a large Japanese general population cohort (45,246 people) [18]. They found a considerably increased risk of suicide in females with high TC (HR = 1.90, 95% CI, 1.13–3.19) compared to women with normal TC. However, a correlation between TC levels and suicide in males was not found. [18]. 

In a study of 288 patients conducted by Ma et al. [47], 20.14% had attempted suicide in the previous month. The authors found lower total cholesterol, and more symptoms of psychosis. However, in their conclusions, these authors indicated that none of these significant results survived following the Bonferroni correction. 

Reuter et al. [48] conducted an interesting study of veterans with suicidal ideation, a documented suicide attempt, or who had committed suicide between 2009 and 2015 and were included in the Suicide Prevention Coordinator database. They demonstrated that veterans with total cholesterol levels below 168 mg/dl had a greater risk for suicide compared to those with higher levels. The cholesterol levels of veterans reporting suicidal ideation or attempted suicide were significantly lower than the group reporting neither of these [F(2, 185) = 30.19, *p* < 0.001] [48]. By contrast, Capuzzi et al. showed that individuals with levels of total cholesterol below 160 mg/dL had lower rates of recent suicide attempts compared to those with values above 160 mg/dL [49]. Suenson et al. also reported negative correlations between serum total cholesterol (TC) and a compound variable of aggressive behavior in those who attempted suicide [40]. Ma et al. (2020) reported that persons who had made suicide attempts had higher total levels of TC compared with persons who had not [50].

### 3.2. Low-Density Lipoprotein

It is suggested that low-density lipoprotein cholesterol (LDL-c) should not exceed 100 milligrams per deciliter (mg/dL). Levels of 100 to 129 mg/dL are normal for individuals without heart diseases. A level of 130 to 159 mg/dL is borderline high and 160 to 189 mg/dL is high. A level of 190 mg/dL or higher is considered very high [41].

Aguglia et al. [44] showed that high lethality suicide attempters had considerably lower LDL compared to low lethality attempters and the control group [44]. 

Segoviano-Mendoza et al. [6] also reported that LDL-c serum levels were lowered significantly in the patients with MDD and a suicide attempt compared to those without suicidal behavior.

Suenson et al. [40] showed negative associations between LDL-c with a compound variable of aggressive state and between LDL-c and a compound variable of aggressive personality traits.

In another study an association was found between the incremental increase of low-density lipoprotein (HR = 1.11, 95% CI, 1.02–1.21) and an increased risk of suicide in females [18].

Among others, Ma et al. found a correlation between lower LDL-c and more psychotic symptoms showed [47].

Su et al. compared blood lipid profile markers between unipolar and bipolar depressed patients and in depressed patients with anhedonia or suicidal thoughts [51]. Patients with bipolar had significantly lower LDL-c. In turn, depressed patients with depression and anhedonia had considerably higher LDL-c levels compared to patients without anhedonia [51].

Additionally, Ayesa-Arriola et al. [52] showed that low levels of LDL-c ((OR = 0.99, 95% CI= 0.98−1.00) and depressive symptoms were correlated with suicidal behavior [52].

In the study conducted by Kavoor et al., which compared patients with schizophrenia with healthy controls, [53] LDL-c levels showed significant negative correlations with scores on impulsivity and suicidality [53].

Shaker et al. focused on the correlation between lipid levels and impulsivity in suicidal patients with major depressive disorder. They found that the LDL-c levels are important factors impacting the incidence of suicide and thus could be predictors for suicidality independent of impulsivity [54].

On the other hand, Ma et al. (2020) showed that suicide attempters had considerably higher levels of LDL-c compared with non-attempters [50].

### 3.3. Very Low-Density Lipoprotein

There are only a few studies that focused on changes of very low-density lipoprotein (VLDL) levels in suicide attempters. In the study conducted by Kavoor et al. [53] there is no correlation between scores on the Beck Scale for suicidal ideation (BSI) and VLDL levels in patients with schizophrenia. In contrast, Segoviano-Mendoza et al. [6] found a significant decrease in VLDL-cholesterol in patients with MDD and suicide attempts compared to those without suicidal behavior.

### 3.4. High Density Lipoprotein

It is suggested that high density lipoprotein (HDL) levels ought to be kept higher. HDL levels below 40 mg/dL are considered a most important risk factor for heart disease. Level from 41 mg/dL to 59 mg/dL is considered borderline low. The optimal range for HDL levels is 60 mg/dL or higher [41].

In the study conducted by Ma et al. suicide attempters had significantly lower high-density lipoprotein cholesterol (HDL-c) levels compared with non-attempters [49].

Similar results were reported by Loas et al., who found that a high level of suicidal ideation was associated with low levels of HDL [54]. 

Shaker et al. also found that low HDL level was significantly associated with suicidality and high suicide intent was associated with hopelessness. [55].

Likewise, suicide attempts were related to decreased HDL-C in the study performed by Zhao et al. [56].

Aguglia et al. [44] compared the low lethality group (LLSA) including 299 subjects with a group of high lethality suicide attempters (HLSA) including 133 subjects. The LLSA group exhibited higher HDL-c levels compared to HLSA group. 

Su et al. had other conclusions [51]. In their study persons with suicidal thoughts had higher levels of HDL as compared with controls.

## 4. Triglycerides

The healthy range of triglycerides are as follows: normal—below 150 mg/dL; borderline high—150 to 199 mg/dL; high—200 to 499 mg/; very high—500 mg/dL or above [41].

In the study conducted by Aguglia, the control group had higher triglyceride levels compared to patients admitted to the psychiatric departments for a suicide attempt [43].

The high level of triglycerides in serum has been related with depression and suicidality. [57]. There was also a significant correlation between the high level of triglycerides (≥150 mg/dL and depression in females.

Shin et al. conducted a study that used data obtained from a representative Korean sample of 4265 people aged 65 years or older. They found that lower triglyceride levels were correlated with a decreased risk of suicidal ideation among males (OR = 0.65; 95% CI = 0.43–0.99) but not among females [58].

Eidan et al. [59] tested whether interleukin-6 (IL−6) and interferon-gamma (INF-γ) levels, as well as lipid profile, are related with suicide attempts in adult patients with major depressive disorder. Triglyceride concentrations were considerably higher in both the group of patients who had attempted suicide and nonsuicide attempt group compared to healthy controls. No significant difference in triglyceride concentrations was observed between the suicide attempt and nonsuicide attempt groups [59].

In contrast, Segoviano-Mendoza et al. [6] obtained different results. They showed a statistically significant connection between hypocholesterolemia and suicide attempt (OR 5.540 CI 95% 2.825–10.866, *p* < 0.001).

## 5. Discussion

It is well known that low levels of cholesterol, triglycerides, and LDL are beneficial in the prevention of cardiovascular diseases. However, some studies presented in our review may indicate that low lipid values may be associated with a higher risk of suicide attempts [6,42,52,58].

Many researchers [6,18,42,43,44,45,46,47,48,49,50,51,52,53,54,55,56,57,58,59,60,61] have attempted to identify biological markers that could be linked to suicidal behavior and potentially be used as additional preventive factors. Some of them tried to find the link between cytokines, such as TNF-α, IL-1β, and IL-6 and suicide [59,60]; others measured plasma phospholipid levels of arachidonic acid (AA%), docosahexaenoic acid (DHA%) and eicosapentaenoic acid (EPA%) [61]. Levels of thyroid thyroxine (T4), triiodothyronine (T3), thyroid-stimulating hormone (TSH), prolactin, and cortisol were also measured [51]. Other researchers [6,18,42,43,44,45,46,47,48,49,50,51,52,53,54,55,56,57,58,59] have focused on the correlation between plasma or serum lipid levels and suicide but, unfortunately, inconsistent results have been reported. Both an increase and a decrease in lipid levels were observed.

We have collected possible variants of changes in the level of lipids (total cholesterol very-low-density lipoprotein, low-density lipoprotein, high-density lipoprotein, and triglycerides) depending on the mental disorder in Table 1.

There are other studies focused on the relationship between psychiatric disorders and serum lipid levels, like attention-deficit/hyperactivity disorder, obsessive-compulsive disorder, anxiety, or personality disorders [39,60,61,62,63,64]. However, in our article we have limited our research to a specific timeframe, adult patients only, and mental illnesses such as depression, bipolar disorder, and schizophrenia.

In 2017, Bartoli et al. [37] published a meta-analysis that included data on lipid profiles from 11 studies based on 288 subjects with histories of attempted suicide and 754 without such attempts. They found no differences in total cholesterol, LDL, or triglycerides. Another meta-analysis performed by Wu et al. showed that lower serum TC levels were associated with a 112% higher risk of suicidality, including a 123% higher risk of suicide attempts and an 85% higher risk of suicide completion [3]. 

The meta-analyses cited above also showed inconsistent results. These discrepancies are because suicide-related behaviors are a complex and multifaceted phenomenon. Support programs, psychotherapy, education about suicidal crises, and cooperation with professionals should be the first line of aid for those who express a desire to commit suicide [65,66].

## 6. Conclusions

Many studies have tried to identify lipids as biological markers of suicidal behavior. However, inconsistent results have been reported. The identification of useful and reliable biological markers of suicide remains a critical need and, thereby, suggests a field for future research.

## Figures and Tables

**Table 1:** Possible variants of changes in the level of lipids in depression, bipolar disorder and schizophrenia.

Psychiatric Disorders	Lpids Levels	Possibility of Suicide
Major depressive disorder	TC ↓ LDL ↓ VLDL ↓ HDL ↓ or ↑ TG ↑	↑ suicide
Bipolar disorder	TC ↑ LDL ↓ VLDL ↓ HDL ↑ TG ↓	↑ suicide
Schizophernia	TC ↓ LDL ↓ VLDL-no changes HDL ↓ TG-no changes	↑ suicide

↓: Decrease; ↑: Increase.

## Data Availability

Data availability on request.
